# A Gain-of-Function Mutation in *Adenylate Cyclase 3* Protects Mice from Diet-Induced Obesity

**DOI:** 10.1371/journal.pone.0110226

**Published:** 2014-10-16

**Authors:** Jeffrey L. Pitman, Matthew C. Wheeler, David J. Lloyd, John R. Walker, Richard J. Glynne, Nicholas Gekakis

**Affiliations:** 1 Department of Cell and Molecular Biology, The Scripps Research Institute, La Jolla, CA, United States of America; 2 Genomics Institute of the Novartis Research Foundation, San Diego, CA, United States of America; 3 Donald P. Shiley BioScience Center, San Diego State University, San Diego, CA, United States of America; University of Texas Health Science Center at Houston, United States of America

## Abstract

In a screen for genes that affect the metabolic response to high-fat diet (HFD), we selected one line of *N*-ethyl-*N*-nitrosourea (ENU)-mutagenized mice, *Jll*, with dominantly inherited resistance to diet-induced obesity (DIO). Mutant animals had dramatically reduced body weight and fat mass, and low basal insulin and glucose levels relative to unaffected controls. Both white adipose tissue (WAT) and brown adipose tissue (BAT) depots were smaller in mutant animals. Mutant animals fed a HFD gained only slightly more weight than animals fed regular chow, and were protected from hepatic lipid accumulation. The phenotype was genetically linked to a 5.7-Mb interval on chromosome 12, and sequencing of the entire interval identified a single coding mutation, predicted to cause a methionine-to-isoleucine substitution at position 279 of the Adcy3 protein (*Adcy3^M279I^*, henceforth referred to as *Adcy3^Jll^*). The mutant protein is hyperactive, possibly constitutively so, producing elevated levels of cyclic AMP in a cell-based assay. These mice demonstrate that increased *Adcy3* activity robustly protect animals from diet-induced metabolic derangements.

## Introduction

Obesity is both a national and worldwide epidemic. The prevalence of obesity (body mass index (BMI) >30) is greater than 30% in the United States, and is even higher in some parts of the world with the highest prevalence in the Pacific Islands [1]. Obesity increases the risk of heart disease, type 2 diabetes, hypertension, stroke, colon cancer, and early mortality [2], [3].

Complex genetic and environmental factors affect the risk of developing obesity. Evidence for a genetic contribution to obesity comes from animal models, monogenic obesity in humans, twin and adoption studies, and genome-wide association studies (GWAS). Single-gene mutations that cause obesity in mice or rats have long been known, establishing clearly that genetic mutations can cause obesity, and elucidating the pathways that control energy homeostasis [4]. Central among these is *Leptin*, which is produced by adipose tissue in proportion to total body fat and acts in the hypothalamus to regulate energy intake and expenditure [5]. Cases of monogenic human obesity have been traced to mutations in *LEPTIN*, genes in the leptin signaling pathway, and other genes not directly implicated in leptin biology [6]. In the case of prohormone convertase 1 (PC1), monogenic obesity was identified in humans before a corresponding, obesity-causing mutation was identified in mice [7], [8]. While these studies establish a genetic cause in rare, severe obesity, evidence for a genetic contribution to common obesity comes from adoption and twin studies, which show that 60–80% of variation in BMI is hereditary [9]. GWAS have identified many known and novel genes that contribute to variation in BMI. The genetic variants identified by GWAS so far, however, account for only a small percentage of the heritability in BMI [10].

One of the obesity loci identified by GWAS maps at or near the *ADCY3* gene, which encodes a member of the adenylate cyclase (AC) family of proteins [11]–[14]. Additional genetic evidence supports an association between *ADCY3* variation and obesity in both Swedish and Han Chinese populations [15], [16]. Animal models have also highlighted the importance of *Adcy3* signaling in energy homeostasis, with *Adcy3* ^-/-^ mice having more fat mass under basal conditions and being more susceptible to obesity induced by high-fat feeding [17].

Proteins of the AC family catalyze the production of cAMP, a second messenger in many signal transduction pathways. Cyclic AMP is involved in the control of energy homeostasis by many neurotransmitters and (neuro)endocrine peptides including α-melanocyte-stimulating hormone (α-MSH), the Orexins, GLP-1, Ghrelin, and others [18], [19]. Signaling from β-adrenergic receptors causes elevated cAMP and subsequent lipolysis in WAT and BAT; mice lacking all three β-adrenergic receptors (β1-, β2-, and β3-AR) have reduced metabolic rate and are slightly obese on chow, and massively obese on HFD [20]. Conversely, β3-adrenergic stimulation by a synthetic ligand promotes energy expenditure and protects against diet-induced obesity in both mice and primates [21]. Cyclic AMP responsive element binding protein (Creb) and Creb-regulated transcription co-activator 1 and 3 (Crtc1 and 3) mediate transcriptional responses to cAMP. Crtc3 is important in attenuating β-adrenergic signaling in adipose tissue as *Crtc3* ^-/-^ mice have an increased response to catecholamine and are resistant to DIO [22]. Crtc1, on the other hand, is required for proper *Leptin* response within the hypothalamus and *Crtc1* ^-/-^ mice fail to up-regulate the anorexigenic peptides Cartpt and Kiss1 in response to leptin, and are hyperphagic and obese [23].

Upon activation of beta-adrenergic receptors, heterotrimeric G-proteins dissociate and the α subunits signal to effector molecules. G_S_α – encoded by the *Gnas* gene - is the stimulatory subunit that activates AC to promote production of cAMP. Deletion specifically of G_S_α leads to mice that are obese and insulin resistant [24]. Knockout of *Adcy3* itself also leads to obesity in mice [17]. cAMP activates cAMP-dependent protein kinase (PKA), which is composed of two regulatory and two catalytic subunits. cAMP binds the regulatory subunits causing their dissociation from the catalytic subunits, which are then activated. Knockout of RIIβ, a regulatory subunit of protein kinase A, leads to increased cAMP signaling and a lean mouse that is resistant to genetic or diet-induced obesity [25], [26].

Thus, human genetics and animal models have converged on cAMP signaling in general, and *Adcy3* in particular, as central players in energy homeostasis. Here we describe a novel allele of *Adcy3*, identified through forward genetic screening of mutagenized mice, which protects mice from diet-induced obesity. No difference in food intake was found between mutant mice and their wild-type littermates, but mutant mice expended more energy. The mutant allele was hyperactive relative to the wild-type allele in generating cAMP in response to forskolin.

## Materials and Methods

### Animals and ENU mutagenesis

This study was carried out in strict accordance with the recommendations in the Guide for the Care and Use of Laboratory Animals of the National Institutes of Health. The protocols were approved by the Institutional Animal Use and Care Committee of the Scripps Research Institute or Genomics Institute of the Novartis Research Foundation. All mice were housed under a standard 12-hour light cycle with free access to food and water. A subset of mice were given a HFD, which derived 60% of its calories from fat (TD.06414, Teklad), beginning at three to five weeks of age and for the duration of the experiment. ENU mutagenesis and breeding of mutant animals have been described [8], [27].

### Histology

Animals at the eighth generation of backcross to the C57BL6/J (B6) background were sacrificed after twelve weeks on HFD (17 weeks of age), or after continued observation of growth at one year of age. Tissues were excised and fixed in formalin for later sectioning and staining with hemotoxylin and eosin. Sectioning and staining were performed by the TSRI Histology core. Adipocyte cross-sectional area was measured by light microscopy of stained sections using ImageJ software with manual selection of cells.

### Mapping and identifying the gene responsible for the *Jll* phenotype

The affected (*Jll*) family was identified in ENU-mutagenized B6 mice and backcrossed to B6 to demonstrate heritability of the *Jll* phenotype. Mice with the *Jll* phenotype were also outcrossed to 129S1/SvIMJ (129) mice and progeny were selected based on NMR-assessed total body fat content for mapping of the *Jll* phenotype, as previously described [28]. Based on a 5.7-Mb interval on chromosome 12 defined by genetic mapping, an interval-spanning capture array of tiled genomic fragments was designed by Nimblegen [29]. Libraries prepared from genomic DNA from a single homozygous *Jll* mutant animal was captured on the Nimblegen array. Captured DNA was sequenced using 454 pyrosequencing [30].

The sequence accession numbers used in murine adenylate cyclase and cross-species Adcy3 ortholog alignments were Mm Adcy1 (gene ID 432530), NP_033752.1; Mm Adcy2 (gene ID 210044), NP_705762.2; Mm Adcy3 (gene ID 104111), NP_612178.2; Mm Adcy4 (gene ID 104110), NP_536683.1; Mm Adcy5 (gene ID 224129), NP_001012783.3; Mm Adcy6 (gene ID 11512), NP_031431.2; Mm Adcy7 (gene ID 11513), NP_001032813.1; Mm Adcy8 (gene ID 11514), NP_033753.2; Mm Adcy9 (gene ID 11515), NP_033754.2; Hs ADCY3 (gene ID 109), NP_004027.2; Rn Adcy3 (gene ID 64508), NP_570135.2; Am Ac3 (gene ID 408363), NP_001071276.1; Dm Ac3 (gene ID 35419), NP_001014496.1.

### Plasmid DNA used and cAMP response assay

Full-length mouse *Adcy3* cDNA was obtained from Origene. A cAMP-responsive element-luciferase reporter gene was obtained from the Montminy lab [31]. Transient transfections of human hepatoma HuH7 cells were performed as follows. Exponentially growing cells were seeded onto a 96-well plate. The following day, cells were transiently transfected with a cocktail of the CRE-luciferase reporter and a pcDNA6-2 × FLAG-tagged expression vector (empty vector, wild-type Adcy3, or Adcy3^Jll^) using Lipofectamine 2000 (Life Technologies). The next day, all wells were switched to 100 µl per well fresh DMEM/10% FBS, plus either DMSO or forskolin (0.2 µM, 1 µM, 5 µM final concentrations). Forskolin was serially diluted in DMSO to 100× final concentration, and the same volume of DMSO or DMSO + forskolin was added to each well. After a five-hour incubation in this media, all wells were harvested and luminescence assessed using BrightGlo luciferase reagent (Promega) and an Acquest luminometer (Molecular Devices).

### qPCR

Tissues were dissected and placed in Trizol (Sigma) on ice, prior to homogenization and RNA preparation according to the manufacturer's instructions. RNA abundance was measured by quantitative PCR, using an ABI 7900 HT, as described [28].

### Body composition analysis

Male and female wild-type and heterozygous *Jll* littermates (N = 2 to 4 in each group), were weaned and placed on HFD at five weeks of age. Body weight and body composition were assessed at two-week intervals from 5 to 17 weeks, or at six- to eight-week intervals from 17 to 54 weeks. Fat and lean mass of live mice was measured by NMR on an EchoMRI 3-in-1. Following completion of body composition analysis at 54 weeks, animals were sacrificed, and fat pads and other tissues were dissected for RNA analysis.

### Plasma metabolite and protein determinations

Animals were fasted for six hours prior to orbital bleed. Blood was collected by retro-orbital bleed and stored on ice in EDTA tubes. Plasma was assessed for leptin, insulin, glucose, and cholesterol levels by ELISA (insulin, leptin), or using an Olympus AU400 Autoanalyzer (glucose and cholesterol) as described [28].

### Whole-animal metabolic measurements (indirect calorimetry)

Animals used were males, either wild-type (N = 7) or *Adcy3^Jll/+^* heterozygous (N = 6), from the sixth generation of outcrossing to 129. Animals were weaned onto regular chow (not HFD) to minimize differences due to obesity. All animals were nine weeks old at the start of the experiment. Animals were housed separately for the experiment, and allowed to acclimate to new cages before the observation period. After an initial three-day acclimation period, observation and measurement lasted seven days, and took place at the TSRI Mouse Behavioral Assessment Core.

### Statistical Analysis

All statistical analyses were done by Student's t-test. In mice, all comparisons were within sex to wild-type mice. In other cases the relevant comparisons are described in the figure legend. Statistically significant differences are noted.

## Results

### *Jll* mutation dominantly suppresses diet-induced obesity

**Figure 1:**
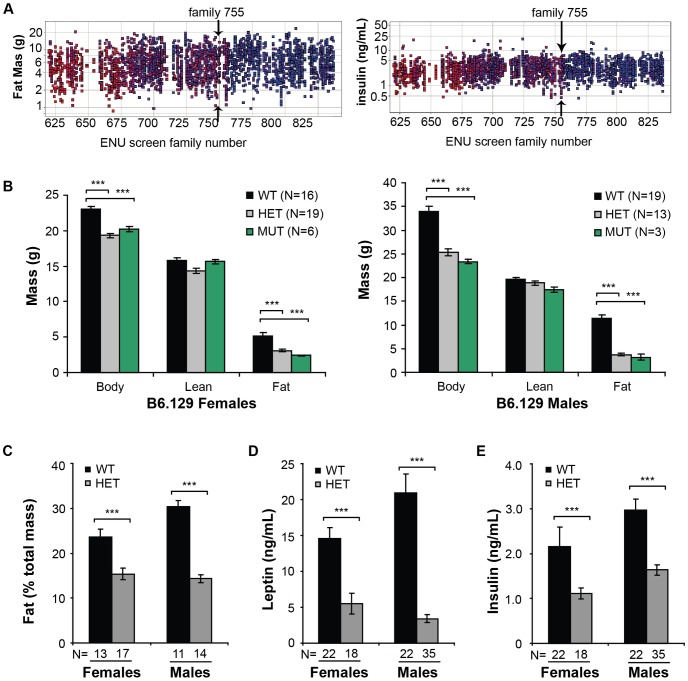
Identification and phenotype of the *Jll* allele. **A**. Individual fat mass (left panel) and insulin (right panel) values for selected mouse families from the ENU screen; family 755 (*Jll*) is marked with arrows. Each square represents a single mouse; individuals within a family are in the same column and are the same color. **B**. Phenotypes for B6.129 *Jll* mice. Genotypes reflect homozygosity for 129-specific allele SNPs (WT, black) heterozygosity for B6 and 129 SNPs (HET, gray), or homozygosity for B6-specific SNPs (MUT, green). Note that mice homozygous for the ENU-induced mutation do not significantly differ from heterozygotes. **C**. Overall percent body fat is lower in *Jll* mutant animals in the B6.129 hybrid background. **D–E**. Fasted plasma leptin (D) and insulin (E) concentrations are significantly lower in 3-month-old male heterozygotes than in their wild-type littermates. The graph is colored and labeled as in (B). All data are shown as mean +/− SEM, ***, p<0.001 by Student's t-test.

The *Jll* phenotype was initially identified by screening families of ENU-mutagenized C57BL/6J mice for changes in body composition or fasting plasma insulin. All animals were placed on HFD at 4 weeks of age and then assessed for total body weight, fat mass, and insulin levels at 12 weeks of age. Family 755 was enriched for mice with low fat mass despite eight weeks of high-fat feeding (Figure 1A, left panel). The same mice also had low fasting insulin levels (Figure 1A, right panel).

Affected mice were either backcrossed to B6, to maintain mutant mice on a pure B6 background with minimal contributions from other ENU-induced mutations, or outcrossed to 129, to map the *Jll* mutation on a hybrid B6.129 background. Since successive litters of outcrossed B6 mice contain roughly 50% affected animals (Figure S1A), the *Jll* phenotype appeared to be dominantly inherited, such that mice heterozygous for the mutation were resistant to HFD-induced obesity.

### *Jll* mutant mice have less fat and improved metabolic parameters

The dominant *Jll* mutation was mapped by outcrossing an affected (resistant to DIO) mouse from family 755 to 129. Outcrossing the affected F1 progeny to 129 generated N2 and N3 mice, which allowed genetic mapping of the mutation. Whole-genome scans were performed on B6.129 N2 and N3 mice, and resistance to DIO was compared to inheritance of B6-specific single nucleotide polymorphisms (SNPs). This revealed a strong correlation between inheritance of the low-fat-mass phenotype and inheritance of B6-specific SNPs from an approximately 5.7- Mb interval of chromosome 12, from 3.9 to 9.6 Mb (Figure S1B). This genomic region defined the minimal *Jll* mutant interval. These SNP markers were then used to tag the mutated locus and allow further characterization of the *Jll* phenotype on the B6.129 hybrid background.

Twelve-week-old *Jll* heterozygous animals had significantly lower body weights and fat mass than did their wild-type siblings (p<0.001, Figure 1B), after eight weeks of high-fat feeding, without a difference detectable in lean mass. Genotype-specific differences in body and fat mass were much larger in males than females. Mice homozygous for the *Jll*-containing mutant interval also had significantly lower total body mass and fat mass than wild-type mice (p<0.001, Figure 1B). Homozygous *Jll/Jll* mice did not significantly differ from *Jll/+* mice in body composition, confirming a dominant phenotype (Figure 1B). We therefore compare *Jll*/+ heterozygous animals to their wild-type counterparts hereafter.

Both males and females heterozygous for *Jll* have substantially reduced percent body fat (fat mass as a percentage of total body mass, p<0.001, Figure 1C). Other metabolic parameters were also notably improved in B6.129 *Jll* mutant mice: fasted plasma leptin levels were dramatically lower in *Jll* heterozygous males (five-fold) and females (three-fold) relative to wild-type at p<0.001 (Figure 1D); and fasted levels of plasma insulin were significantly lower in *Jll*/+ animals than in their wild-type counterparts (p<0.001, Figure 1E). Despite the near-50% reduction in insulin levels, however, fasted plasma glucose levels were slightly lower in *Jll* heterozygous males versus wild-type males (p<0.05), and no difference was detectable in females (Figure S2A). No significant changes in plasma triglycerides, total cholesterol, or HDL cholesterol concentrations were detectable in *Jll* heterozygotes (Figures S2B–D).

### *Jll* is a Met-to-Ile mutation in *Adcy3*

Since the inheritance of the B6 alleles at the chromosome 12 locus reproducibly associated with the *Jll* mutant phenotype, we analyzed genomic DNA from a mouse homozygous for B6 markers across this interval, using next-generation sequencing to assess single base changes across the entire 5.7-Mb *Jll* region. Sequencing reads were then compared to GenBank reference genomic sequence to identify potential mutations. Table 1 lists all sequence variants present in at least five total reads, in which departure from reference sequence was present in at least 75% of reads.

**Table 1:** Sequence variants in *Jll* interval with five or more reads and greater than 75% frequency.

Variant position	Reference nucleotide	Variant nucleotide	Total reads	Variant frequency	Reference amino acid	Variant amino acid	Location
Chrom.12: 3964446	A	G	17	94%	-	-	3′ UTR, *Efr3B*
Chrom.12: 4194328	G	A	10	100%	M	I	exon 3, *Adcy3*
Chrom.12: 4436043	-	C	15	100%	-	-	intron 1, *Ncoa1*
Chrom.12: 4440044	G	-	9	100%	-	-	intron 1, *Ncoa1*
Chrom.12: 4444750	ATT	TG	11	100%	-	-	intron 1, *Ncoa1*
Chrom.12: 4458743	A	G	9	89%	-	-	intron 1, *Ncoa1*
Chrom.12: 5156510	-	C	8	87%	-	-	intron 2, *Klhl29*
Chrom.12: 5236101	A	G	24	100%	-	-	intron 2, *Klhl29*
Chrom.12: 5875920	A	G	14	93%	-	-	intron 1, *LOC100042095*
Chrom.12: 7584149	A	T	9	89%	-	-	400 kb upstream of *ApoB*
Chrom.12: 8919159	T	A	14	100%	-	-	10 kb upstream of *Laptm4a*

**Figure 2:**
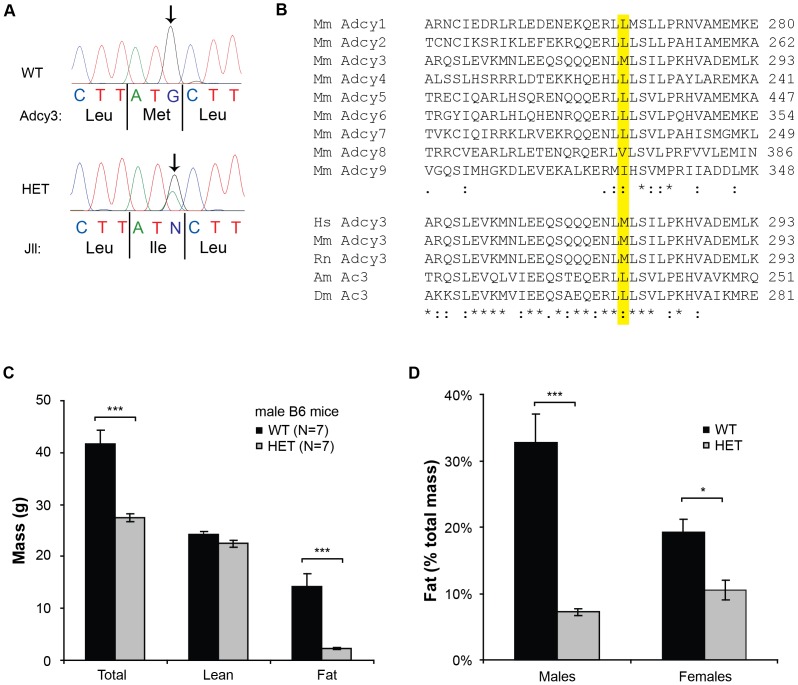
*Jll* is *Adcy3^M279I^*. **A.** Trace files from *Adcy3* exon 3 sequencing of PCR products amplified from wild-type (WT, upper trace) and heterozygous *Jll* mutant (HET, lower trace) genomic DNA. The resulting amino acid sequences of wild-type and mutant Adcy3 proteins (amino acids 278–280) are shown below the traces. Mutant position is marked with arrows. **B.** Alignment of all nine murine Adcy proteins in the region surrounding the *Jll* mutation (upper panel), and conservation of the affected methionine residue in vertebrate Adcy3 orthologs, but not in invertebrate ones (lower panel). Dots reflect weak amino acid similarity, colons similarity, and asterisks identity. Abbreviations used: Mm, mouse; Hs, human; Rn, rat; Am, honeybee; Dm, fruit fly. **C–D.** Mice heterozygous for *Adcy3^M279I^* in a backcrossed B6 background and fed a HFD display the expected *Jll* phenotypes. Male mice have lower body weight and fat mass (C), and both sexes show decreased body fat percent after 24 weeks on a HFD (D). All data are shown as mean +/− SEM, * p<0.05, *** p<0.001 by Student's t-test.

Eleven variants meeting this standard were present, of which only one was present in protein-coding sequence, a G-to-A transition at base 827 of mouse *Adcy3* (Table 1). This resulted in a methionine-to-isoleucine change at amino acid 279 (Figure 2A), which mapped to a relatively conserved region among mouse adenylate cyclases (Figure 2B, upper panel). The analogous region has been shown to be important for activity in other ACs. Met279 sits between the sixth transmembrane domain and the first catalytic C1 region, lying 10 amino acids amino-terminal to a known G_S_α -contacting residue [32]. Surprisingly, the particular amino acid at this position is not absolutely conserved among distant Adcy3 orthologs: while proximal amino acids are mostly invariant, and while Met279 is conserved in other vertebrates, both honeybees (*Apis mellifera*) and fruit flies (*Drosophila melanogaster*) have a leucine at this position (Figure 2B, lower panel).

In order to verify that this mutation does, in fact, confer the *Jll* phenotype, body composition was measured in mice from the original (B6 background) family 755, that had been backcrossed to B6 for five generations. When maintained on HFD, mice heterozygous for the *Adcy3^M279I^* mutation had significantly lower total mass (p<0.001), absolute fat mass (p<0.001), and relative fat mass (p<0.001 for males and p<0.05 for females) than their wild-type littermates (Figure 2C, D). This is true for both males and females, and is indistinguishable from the *Jll* phenotype observed in the mixed B6.129 background. The *Adcy3^M279I^* mutation is therefore henceforth referred to as *Adcy3^Jll^*.

### *Jll* mice do not expand their adipose tissue in response to HFD

**Figure 3:**
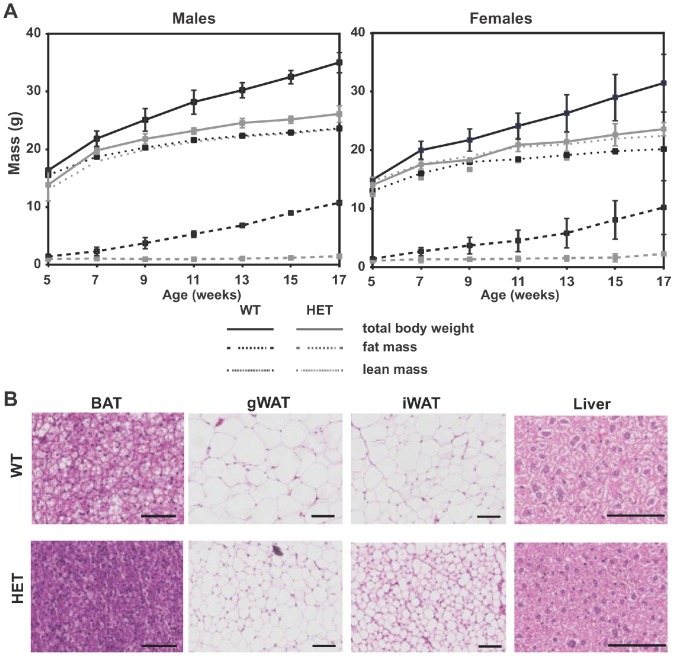
Reduced weight and adipocyte volume in *Jll* mice. **A.** Over a 12-week observation period of age-matched, littermate B6 animals placed on a HFD at five weeks of age, heterozygous *Adcy3^Jll/+^* (HET) mice (gray lines) gained significantly less total body weight (solid lines) and fat mass (dashed lines) than WT controls (black lines), both in male (left panel) and female animals (right panel). In contrast, no significant differences between WT and HET animals were observed in lean mass (dotted lines) over the course of observation, at least in males. All data are shown as mean +/− SEM. **B.** Comparison of paraffin-embedded, hematoxylin- and eosin-stained sectioned wild-type (WT, top row) and heterozygous *Jll* mutant (HET, bottom row) adipose tissues and liver, after twelve weeks of HFD feeding. Abbreviations: BAT, brown adipose tissue; gWAT, gonadal WAT fat pad; iWAT, inguinal WAT fat pad. Scale bars represent 100 µm.

To investigate the dynamic changes in body composition between *Adcy3^+/+^* and *Adcy3^Jll/+^* mice during growth, we placed mice on HFD for twelve weeks beginning at five weeks of age. Mice normally gain lean mass during growth and when challenged with HFD, male and female *Adcy3* ^+/+^ also rapidly gained fat mass (Figure 3A). Their *Adcy3^Jll/+^* littermates however, did not accrue fat mass despite an almost identical gain in lean mass. Both male and female *Adcy3^Jll/+^* mice had significantly lower total body weights and fat mass, compared to wild-type controls, after as little as four weeks on HFD. These differences grew even more pronounced as the 12-week-HFD analysis period progressed, with the fat mass in wild-type animals increasing more than 10-fold, while that in *Adcy3^Jll/+^* mice remained virtually unchanged (Figure 3A). The difference in fat mass was also evident in dissected gonadal (gWAT, p<0.001) and inguinal (iWAT, p<0.01) fat pads (Figure S3B).

Cells in interscapular BAT were smaller in mutant than in wild-type mice, presumably due to a lesser accumulation of lipid on HFD (Figure 3B). A similar reduction of cross-sectional adipocyte area was also observed in heterozygous *Jll* white adipocytes from both the gonadal (p<0.001) and inguinal (p<0.01) white fat depots (Figure 3B and Figure S3C). We did not see evidence for ‘browning’ of WAT in mutant mice by either histology, or by qPCR analysis of *Ucp1*, *Dio2* or *Pgc1a* (Figure S3D). While there is a slight trend toward increased expression of the BAT-specific genes *Ucp1* and *Dio2* in gWAT and iWAT, this trend does not reach statistical significance and is not apparent for *Pgc1a*.

Finally, hepatic steatosis is common in mice maintained on HFD for extended periods, and hematoxylin and eosin staining showed liver pathology consistent with steatosis in WT males after extensive HFD feeding (Figure 3B). In contrast, HET littermates were largely protected against steatosis (Figure 3B, right-most panels).

### Whole-body metabolic measurements: *Jll* mice are more active and expend more energy

**Figure 4:**
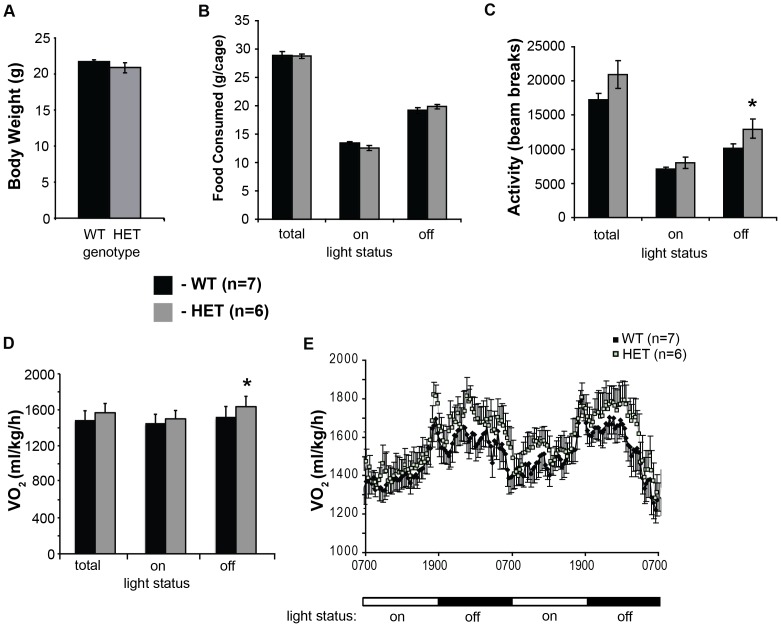
Increased oxygen consumption in *Jll* mice. **A.** Young, male, chow-fed B6.129 N6 animals were used in this experiment, to minimize activity differences resulting from obesity. No significant difference in body weight between *Adcy3^+/+^* (WT, black) and *Adcy3^Jll/+^* (HET, gray) mice was detectable. **B.** Both sets of animals consumed similar amounts of food during the seven days of the experiment. **C.** Increased movement in HET animals was visible, only in the dark phase. **D.** HET animals consumed more oxygen during the dark phase than WT animals. **E.** The increased level of O_2_ consumption (VO_2_) in HET animals, particularly in the dark, is visible in this 48-hour observation period. All data are shown as mean +/− SEM, * p<0.05 by one-tailed Student's t-test.

To better understand the effects of the *Jll* mutation on overall metabolism, we analyzed a total of 13 mice (seven wild-type and six *Adcy3^Jll/+^* heterozygous animals) for seven days using the Oxymax Comprehensive Live Animal Monitoring System. We chose young, age-matched animals that had been raised on chow, in an attempt to minimize changes in activity resulting from obesity, and better focus on the primary effects of the *Jll* mutation. The animals chosen did not differ significantly in overall body weight (Figure 4A), nor did we find a difference in food consumption between *Adcy3* ^+/+^ and *Adcy3^Jll/+^* mice (Figure 4B). There was however a small, but statistically significant, difference in activity that was detectable only during the dark phase (p<0.05, Figure 4C). This may explain the increased energy expenditure (VO_2_) we observed in *Adcy3^Jll/+^* compared to *Adcy3* ^+/+^ mice, which was also detectable only in the dark phase (Figure 4D). This trend is more apparent when measured over a 48-hour period (p<0.05, Figure 4E).

### *Adcy3^Jll^* encodes a gain-of-function adenylate cyclase

**Figure 5:**
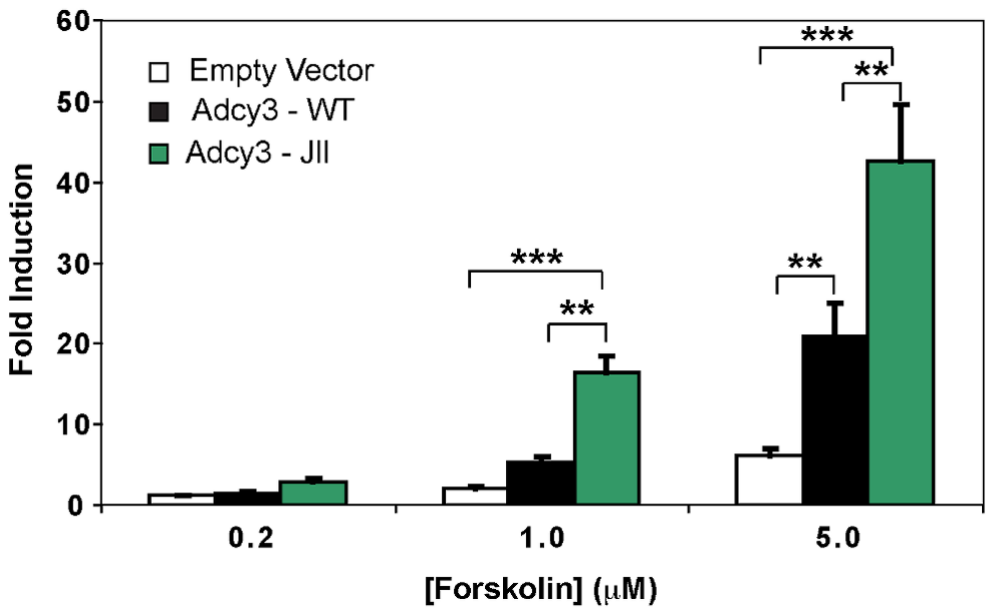
*Jll* expression leads to increased Adcy3 signaling. Treatment of HuH7 cells transiently co-transfected with both a cAMP-responsive luciferase reporter and an empty expression vector (Empty vector), a wild-type mouse *Adcy3* cDNA expression vector (Adcy3-WT), or the same vector with a *Jll* mutation substituted (Adcy3-Jll), revealed increased reporter activity in response to increasing doses of forskolin. For each expression vector used, activity was normalized to the level observed when cells were treated with vehicle (DMSO) alone. All data are shown as mean +/− SEM. ** - p<0.01, *** - p<0.001.

Adenylate cyclase is activated by G_S_α, which promotes the interaction of the cytoplasmic 1 and 2 domains (C_1_ and C_2_, respectively). Forskolin is a small-molecule activator of AC and acts similarly to G_S_α, in that both forskolin and G_S_α promote the association of the C_1_ and C_2_ domains [32]. The *Jll* mutation (M279I) is just upstream of C_1_, near the binding sites for G_S_α and forskolin. The functional activity of wild-type and *Jll* alleles of *Adcy3* in response to forskolin were measured in a reporter gene assay in which wild-type or mutant *Adcy3* expression plasmids were co-transfected into HuH7 cells along with a cAMP responsive element- (CRE-) luciferase plasmid. Luciferase activity was used as a stand in for cAMP production. At low forskolin concentration (0.2 µM), cAMP production by Adcy3^Jll^ was slightly greater than that of Adcy3^+^ (p<0.01), which in turn was similar to cells transfected with empty expression vector (Figure 5). Forskolin increases cAMP production in cells transfected with any of the three expression constructs, but cells expressing Adcy3^Jll^ are more responsive to forskolin than cells expressing Adcy3^+^ (p<0.01), demonstrating higher activity of the *Jll* allele, and consistent with the dominant nature of this allele.

## Discussion

We have identified a dominant, gain-of-function point mutation in a component of the cAMP signaling pathway that promotes whole-body energy metabolism. Mice carrying one or more *Adcy3^Jll^* mutant alleles are protected from high fat diet-induced obesity, are more active, and have lower circulating leptin and insulin levels. We did not detect a difference in plasma TG between WT and HET mice on HFD, suggesting that VLDL production by the liver is normal and supporting the idea that the difference between genotypes is in lipid catabolism. The *Adcy3^Jll^* mutation, though coding for a conservative amino acid substitution, increases Adcy3 activity in response to forskolin.

Following ligand engagement of a G protein-coupled receptor, cAMP acts as a second messenger when AC is activated by G_S_α. Cyclic AMP then activates cAMP-dependent protein kinase (PKA), which phosphorylates many substrates that affect energy, glucose, and lipid metabolism. The energy metabolism pathways affected by cAMP include α-MSH, MCH, and the β-adrenergic pathway, all of which are important intermediates in leptin signaling in the hypothalamus. While *Adcy3* expression is highest in BAT, it is also expressed in several regions of the brain, including hypothalamus. Furthermore, *Jll* mice show no clear expansion of or increased activity – at the level of *Ucp1* or *Dio2* induction in BAT and no “browning” of WAT (Figure 3 and Figure S3). The site of action of *Adcy3^Jll^* therefore remains unresolved, though BAT and/or hypothalamus are likely candidates.

Uncertainty over the critical affected organ is also apparent for *Prkar2b* knockout mice, which, like *Adcy3^Jll^* mice, are lean and protected from genetic and diet-induced obesity [25], [26]. *Prkar2b* encodes RIIβ, which is a regulatory subunit of PKA. Its knockout leads to an isoform switch from type II to type I PKA, which binds cAMP more avidly and is more readily activated. Ucp1 is induced in *Prkar2b* knockout mice and metabolic rate and temperature are increased [25]. Double knockout mice, however, lacking both RIIβ and Ucp1, retain the lean, resistant-to-DIO phenotype [33]. Ucp1 therefore is not necessary for the phenotype and BAT may not be involved. Indeed, re-expression of RIIβ in neurons but not adipocytes reversed the resistance-to-DIO phenotype, indicating that the brain is the critical site of cAMP signaling in *Prkar2b* knockout mice [34].

*Adcy3* ^-/-^ mice are obese because mice eat more and are less active than their wild-type littermates [17]. Underlying the reduced activity and increased appetite may be the reduced response to injected leptin, which leads to appetite suppression and weight loss in wild-type mice, but not in *Adcy3* ^-/-^ littermates. In turn, this reduced leptin response may be attributed to reduced AC activity, which is apparent in hypothalami of *Adcy3* ^-/-^ compared to hypothalami of wild-type mice; even more apparent is reduced AC activity in response to forskolin. In the ventral medial hypothalamus, AC is expressed in primary cilia and its absence may be the reason for a lack of response to leptin [17]. Of course, none of this excludes a role for *Adcy3* in BAT or other tissues and tissue-specific knockouts will be needed to determine the precise site of action of *Adcy3* ^Jll^.

In mammals, there are nine *Adcy* family members, each of which is composed of homologous amino- and carboxy-terminal portions, and each homologous half contains a cluster of six membrane-spanning domains and a large cytoplasmic loop, called C_1_ or C_2_ [35]. Enzymatic activity requires association of the C_1_ and C_2_ domains, which normally have a low affinity for one another. Both forskolin and GTP-bound G_S_α promote the association of C_1_ and C_2_; GTP-G_S_α also alters the shape of the active site to promote catalysis and this may be why forskolin and G_S_α act synergistically [32]. The *Jll* mutation (M279I) lies in C_1_, just upstream of the cyclase catalytic domain and this region was not included in in vitro activity assays or in the crystal structures [32]. Given its proximity to G_S_α and forskolin binding sites however, one would speculate that it affects binding of one or both of these activators.

Human genetics and gain- and loss-of-function alleles in the mouse have identified *Adcy3* as a central, non-redundant player in energy homeostasis. The challenges that now remain include elucidating the precise site of action and mechanism of *Adcy3* that make it an important contributor to obesity or resistance to obesity, identifying the exact genetic variation in human populations that contributes to or protects from obesity, and designing pharmacological interventions based on *Adcy3* that promote fitness. While isoform-specific activators or inhibitors of Adcy3 seem unlikely as the active site is highly conserved, a specific inhibitor has recently been identified [36]. It has also recently become apparent that this family of enzymes is subject to regulation by allosteric effectors [37]. One or more of these approaches may allow Adcy3-targeted therapies to slow the obesity epidemic.

## Supporting Information

Figure S1 **Discovery of** ***Jll*** **.** **A:** Sustained detection of affected mice in outcrossed *Jll* litters. Outcrossing of *Jll* (Family 755) to the 129 background results in approximately half the progeny with low fat mass (2.5 g or below), at both the second generation of outcrossing (N2) and the fifth (N5). In contrast, a broad distribution of fat masses is observed in N2 progeny when wild-type (non-ENU-treated) B6 animals are outcrossed to 129 (Control). **B:** Genome-wide SNP analysis reveals the interval containing *Jll* on mouse chromosome 12. Inheritance of one copy of B6-specific SNPs between 3912084 Mb and 9569145 Mb on mouse chromosome 12 correlates with the *Jll* low fat (fat mass <5 g) and low % fat (below 20%) phenotypes. Living individual animals were phenotyped for fat and lean mass, insulin, and glucose. Genomic tail DNA was extracted and sequenced for an array of SNPs that diverge between the B6 and 129 backgrounds, to detect inheritance from either the ENU-mutagenized (B6) and outcross stock (129) parents. SNPs homozygous for 129 markers are shown in red and marked “C,” SNPs that showed hererozygosity for B6 and 129 markers are shown in orange and marked “H.” “Affected” (aff) and “unaffected” (unaff) phenotypic calls were made based on fat mass, then affected animals were sorted to be on the left of the table, to assist visualizing the genomic interval. The flanking boundaries of the interval are marked with heavy lines.
(TIF)

Figure S2 **Assessment of additional metabolic parameters in** ***Jll*** **mice.** Plasma was prepared from retro-orbital blood of wild-type (WT) and heterozygous *Adcy3^Jll/+^* (HET) mice fed a HFD, and assessed for levels of other metabolically relevant markers. A modest decrease in fed glucose levels (A) was detectable in HET males, but not in females. No significant differences between WT and HET animals of either gender were observed for levels of triglycerides (B), total cholesterol (C) or HDL-cholesterol (D). All data are shown as mean +/− SEM, * p<0.05.
(TIF)

Figure S3 ***Jll*** **protects mice from weight gain on HFD.** **A.** Differences in size are visible between heterozygous *Adcy3^Jll/+^* mice (left) and wild-type (right). **B.** Dissection and quantitation of white fat pad mass showed significantly less massive fat depots in heterozygous *Adcy3^Jll/+^* (HET) mice relative to WT controls, both in gonadal (gWAT) and inguinal (iWAT) depots. **C.** Cross-sectional area of adipocytes from *Adcy3^Jll/+^* (HET) and *Adcy3^+/+^* (WT) mice. For each depot, area was measured with ImageJ software in four mice per genotype and two sections per mouse. Shown are the average ± S.E.M. for each genotype. **D.** Despite the reduction in adipocyte size in *Jll* mutant mice, no dramatic changes in the transcriptional levels of BAT-specific markers, such as *Ucp1*, *Dio2*, and *Pgc1a*, were detected in those adipose depots, N = 3 male mice per tissue per genotype. All data are shown as mean +/− SEM. For **B** and **C**, ** - p<0.01 and *** - p<0.001.
(TIFF)

## References

[1] Nguyen DM, El-Serag HB (2010) The epidemiology of obesity. Gastroenterol Clin North Am 39: 1–7. 20202574 10.1016/j.gtc.2009.12.014 PMC2833287

[2] McGee DL, Diverse Populations C (2005) Body mass index and mortality: a meta-analysis based on person-level data from twenty-six observational studies. Ann Epidemiol 15: 87–97. 15652713 10.1016/j.annepidem.2004.05.012

[3] Field AE, Coakley EH, Must A, Spadano JL, Laird N, et al (2001) Impact of overweight on the risk of developing common chronic diseases during a 10-year period. Arch Intern Med 161: 1581–1586. 11434789 10.1001/archinte.161.13.1581

[4] Leibel RL, Chung WK, Chua SC Jr (1997) The molecular genetics of rodent single gene obesities. J Biol Chem 272: 31937–31940. 9405382 10.1074/jbc.272.51.31937

[5] Friedman JM (2009) Leptin at 14 y of age: an ongoing story. Am J Clin Nutr 89: 973S–979S. 19190071 10.3945/ajcn.2008.26788B PMC2667654

[6] O'Rahilly S (2009) Human genetics illuminates the paths to metabolic disease. Nature 462: 307–314. 19924209 10.1038/nature08532

[7] Jackson RS, Creemers JW, Ohagi S, Raffin-Sanson ML, Sanders L, et al (1997) Obesity and impaired prohormone processing associated with mutations in the human prohormone convertase 1 gene. Nat Genet 16: 303–306. 9207799 10.1038/ng0797-303

[8] Lloyd DJ, Bohan S, Gekakis N (2006) Obesity, hyperphagia and increased metabolic efficiency in Pc1 mutant mice. Hum Mol Genet 15: 1884–1893. 16644867 10.1093/hmg/ddl111

[9] Hanoune J, Defer N (2001) Regulation and role of adenylyl cyclase isoforms. Annu Rev Pharmacol Toxicol 41: 145–174. 11264454 10.1146/annurev.pharmtox.41.1.145

[10] Xia Q, Grant SF (2013) The genetics of human obesity. Ann N Y Acad Sci 1281: 178–190. 23360386 10.1111/nyas.12020 PMC3717174

[11] Speliotes EK, Willer CJ, Berndt SI, Monda KL, Thorleifsson G, et al (2010) Association analyses of 249,796 individuals reveal 18 new loci associated with body mass index. Nat Genet 42: 937–948. 20935630 10.1038/ng.686 PMC3014648

[12] Wen W, Cho YS, Zheng W, Dorajoo R, Kato N, et al (2012) Meta-analysis identifies common variants associated with body mass index in east Asians. Nat Genet 44: 307–311. 22344219 10.1038/ng.1087 PMC3288728

[13] Monda KL, Chen GK, Taylor KC, Palmer C, Edwards TL, et al (2013) A meta-analysis identifies new loci associated with body mass index in individuals of African ancestry. Nat Genet 45: 690–696. 23583978 10.1038/ng.2608 PMC3694490

[14] Cousminer DL, Berry DJ, Timpson NJ, Ang W, Thiering E, et al (2013) Genome-wide association and longitudinal analyses reveal genetic loci linking pubertal height growth, pubertal timing and childhood adiposity. Hum Mol Genet 22: 2735–2747. 23449627 10.1093/hmg/ddt104 PMC3674797

[15] Nordman S, Abulaiti A, Hilding A, Langberg EC, Humphreys K, et al (2008) Genetic variation of the adenylyl cyclase 3 (AC3) locus and its influence on type 2 diabetes and obesity susceptibility in Swedish men. Int J Obes (Lond) 32: 407–412. 17895882 10.1038/sj.ijo.0803742

[16] Wang H, Wu M, Zhu W, Shen J, Shi X, et al (2010) Evaluation of the Association between the AC3 Genetic Polymorphisms and Obesity in a Chinese Han Population. PLoS ONE 5: e13851. 21079816 10.1371/journal.pone.0013851 PMC2973974

[17] Wang Z, Li V, Chan GC, Phan T, Nudelman AS, et al (2009) Adult type 3 adenylyl cyclase-deficient mice are obese. PLoS ONE 4: e6979. 19750222 10.1371/journal.pone.0006979 PMC2735775

[18] Xu TR, Yang Y, Ward R, Gao L, Liu Y (2013) Orexin receptors: Multi-functional therapeutic targets for sleeping disorders, eating disorders, drug addiction, cancers and other physiological disorders. Cell Signal. 10.1016/j.cellsig.2013.07.025 23917208

[19] Wheeler MB, Lu M, Dillon JS, Leng XH, Chen C, et al (1993) Functional expression of the rat glucagon-like peptide-I receptor, evidence for coupling to both adenylyl cyclase and phospholipase-C. Endocrinology 133: 57–62. 8391428 10.1210/endo.133.1.8391428

[20] Bachman ES, Dhillon H, Zhang CY, Cinti S, Bianco AC, et al (2002) betaAR signaling required for diet-induced thermogenesis and obesity resistance. Science 297: 843–845. 12161655 10.1126/science.1073160

[21] Nagase I, Yoshida T, Kumamoto K, Umekawa T, Sakane N, et al (1996) Expression of uncoupling protein in skeletal muscle and white fat of obese mice treated with thermogenic beta 3-adrenergic agonist. J Clin Invest 97: 2898–2904. 8675704 10.1172/JCI118748 PMC507386

[22] Song Y, Altarejos J, Goodarzi MO, Inoue H, Guo X, et al CRTC3 links catecholamine signalling to energy balance. Nature 468: 933–939. 21164481 10.1038/nature09564 PMC3025711

[23] Altarejos JY, Goebel N, Conkright MD, Inoue H, Xie J, et al (2008) The Creb1 coactivator Crtc1 is required for energy balance and fertility. Nat Med 14: 1112–1117. 18758446 10.1038/nm.1866 PMC2667698

[24] Chen M, Gavrilova O, Liu J, Xie T, Deng C, et al (2005) Alternative Gnas gene products have opposite effects on glucose and lipid metabolism. Proc Natl Acad Sci U S A 102: 7386–7391. 15883378 10.1073/pnas.0408268102 PMC1129092

[25] Cummings DE, Brandon EP, Planas JV, Motamed K, Idzerda RL, et al (1996) Genetically lean mice result from targeted disruption of the RII beta subunit of protein kinase A. Nature 382: 622–626. 8757131 10.1038/382622a0

[26] Newhall KJ, Cummings DE, Nolan MA, McKnight GS (2005) Deletion of the RIIbeta-subunit of protein kinase A decreases body weight and increases energy expenditure in the obese, leptin-deficient ob/ob mouse. Mol Endocrinol 19: 982–991. 15618289 10.1210/me.2004-0343

[27] Lloyd DJ, Hall FW, Tarantino LM, Gekakis N (2005) Diabetes insipidus in mice with a mutation in aquaporin-2. PLoS Genet 1: e20. 16121255 10.1371/journal.pgen.0010020 PMC1189073

[28] Lloyd DJ, Wheeler MC, Gekakis N (2010) A Point Mutation in Sec61{alpha}1 Leads to Diabetes and Hepatosteatosis in Mice. Diabetes 59: 460–470. 19934005 10.2337/db08-1362 PMC2809972

[29] Choi M, Scholl UI, Ji W, Liu T, Tikhonova IR, et al (2009) Genetic diagnosis by whole exome capture and massively parallel DNA sequencing. Proc Natl Acad Sci U S A 106: 19096–19101. 19861545 10.1073/pnas.0910672106 PMC2768590

[30] Margulies M, Egholm M, Altman WE, Attiya S, Bader JS, et al (2005) Genome sequencing in microfabricated high-density picolitre reactors. Nature 437: 376–380. 16056220 10.1038/nature03959 PMC1464427

[31] Conkright MD, Guzman E, Flechner L, Su AI, Hogenesch JB, et al (2003) Genome-wide analysis of CREB target genes reveals a core promoter requirement for cAMP responsiveness. Mol Cell 11: 1101–1108. 12718894 10.1016/s1097-2765(03)00134-5

[32] Tesmer JJ, Sunahara RK, Gilman AG, Sprang SR (1997) Crystal structure of the catalytic domains of adenylyl cyclase in a complex with Gsalpha.GTPgammaS. Science 278: 1907–1916. 9417641 10.1126/science.278.5345.1907

[33] Nolan MA, Sikorski MA, McKnight GS (2004) The role of uncoupling protein 1 in the metabolism and adiposity of RII beta-protein kinase A-deficient mice. Mol Endocrinol 18: 2302–2311. 15192081 10.1210/me.2004-0194

[34] Zheng R, Yang L, Sikorski MA, Enns LC, Czyzyk TA, et al (2013) Deficiency of the RIIbeta subunit of PKA affects locomotor activity and energy homeostasis in distinct neuronal populations. Proc Natl Acad Sci U S A 110: E1631–1640. 23569242 10.1073/pnas.1219542110 PMC3637730

[35] Hurley JH (1999) Structure, mechanism, and regulation of mammalian adenylyl cyclase. J Biol Chem 274: 7599–7602. 10075642 10.1074/jbc.274.12.7599

[36] Conley JM, Brand CS, Bogard AS, Pratt EP, Xu R, et al (2013) Development of a high-throughput screening paradigm for the discovery of small-molecule modulators of adenylyl cyclase: identification of an adenylyl cyclase 2 inhibitor. J Pharmacol Exp Ther 347: 276–287. 24008337 10.1124/jpet.113.207449 PMC3807067

[37] Seifert R, Beste KY (2012) Allosteric regulation of nucleotidyl cyclases: an emerging pharmacological target. Sci Signal 5: pe37. 22949734 10.1126/scisignal.2003466

