# Supplementary figures and images for: A Gain-of-Function Mutation in Adenylate Cyclase 3 Protects Mice from Diet-Induced Obesity
Source: PLoS One. 2014 Oct 16;9(10):e110226. doi: 10.1371/journal.pone.0110226 (PMC4199629; doi:10.1371/journal.pone.0110226)

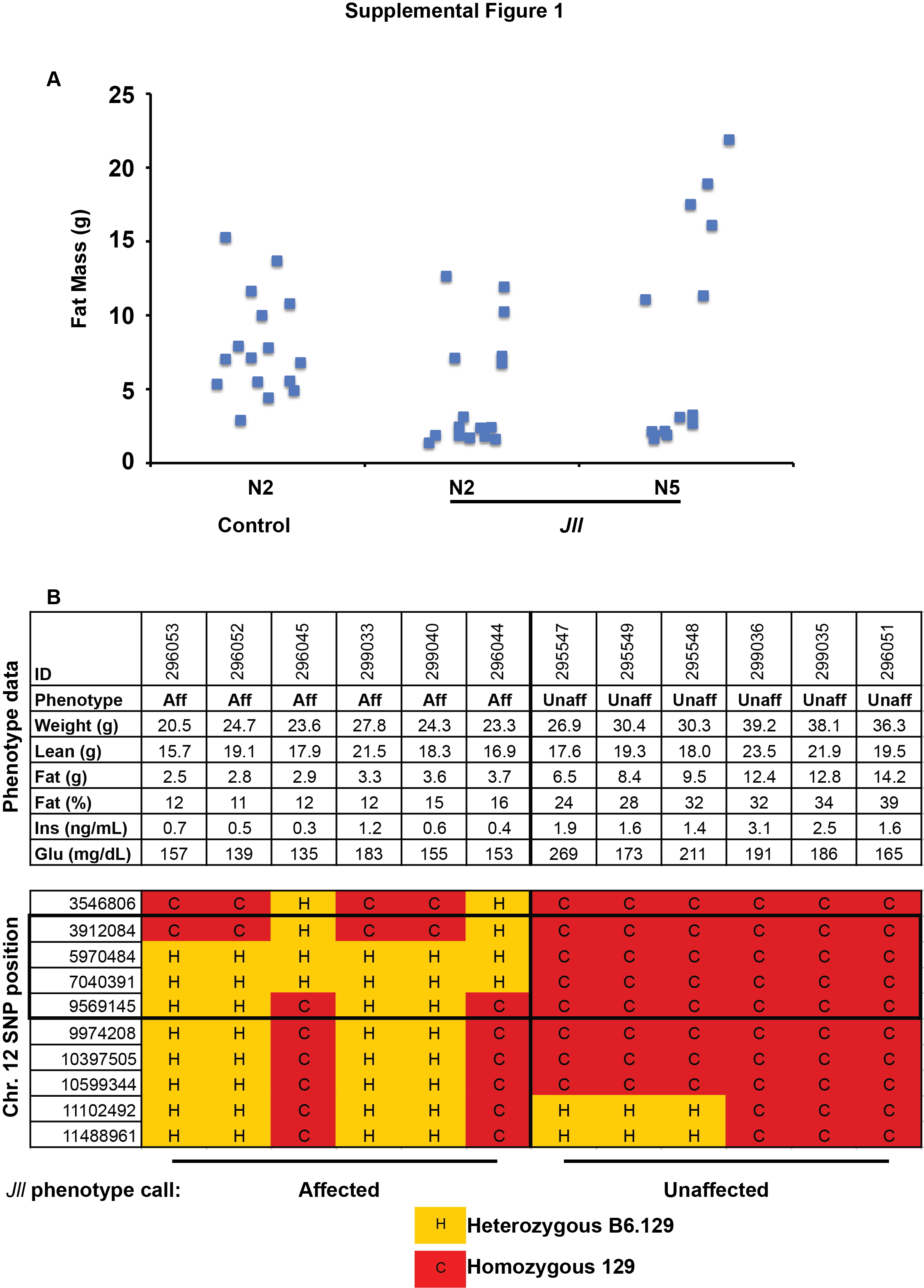

Supplement: Figure S1 — Discovery of Jll . A: Sustained detection of affected mice in outcrossed Jll litters. Outcrossing of Jll (Family 755) to the 129 background results in approximately half the progeny with low fat mass (2.5 g or below), at both the second generation of outcrossing (N2) and the fifth (N5). In contrast, a broad distribution of fat masses is observed in N2 progeny when wild-type (non-ENU-treated) B6 animals are outcrossed to 129 (Control). B: Genome-wide SNP analysis reveals the interval containing Jll on mouse chromosome 12. Inheritance of one copy of B6-specific SNPs between 3912084 Mb and 9569145 Mb on mouse chromosome 12 correlates with the Jll low fat (fat mass <5 g) and low % fat (below 20%) phenotypes. Living individual animals were phenotyped for fat and lean mass, insulin, and glucose. Genomic tail DNA was extracted and sequenced for an array of SNPs that diverge between the B6 and 129 backgrounds, to detect inheritance from either the ENU-mutagenized (B6) and outcross stock (129) parents. SNPs homozygous for 129 markers are shown in red and marked “C,” SNPs that showed hererozygosity for B6 and 129 markers are shown in orange and marked “H.” “Affected” (aff) and “unaffected” (unaff) phenotypic calls were made based on fat mass, then affected animals were sorted to be on the left of the table, to assist visualizing the genomic interval. The flanking boundaries of the interval are marked with heavy lines. (TIF) [file pone.0110226.s001.tif]

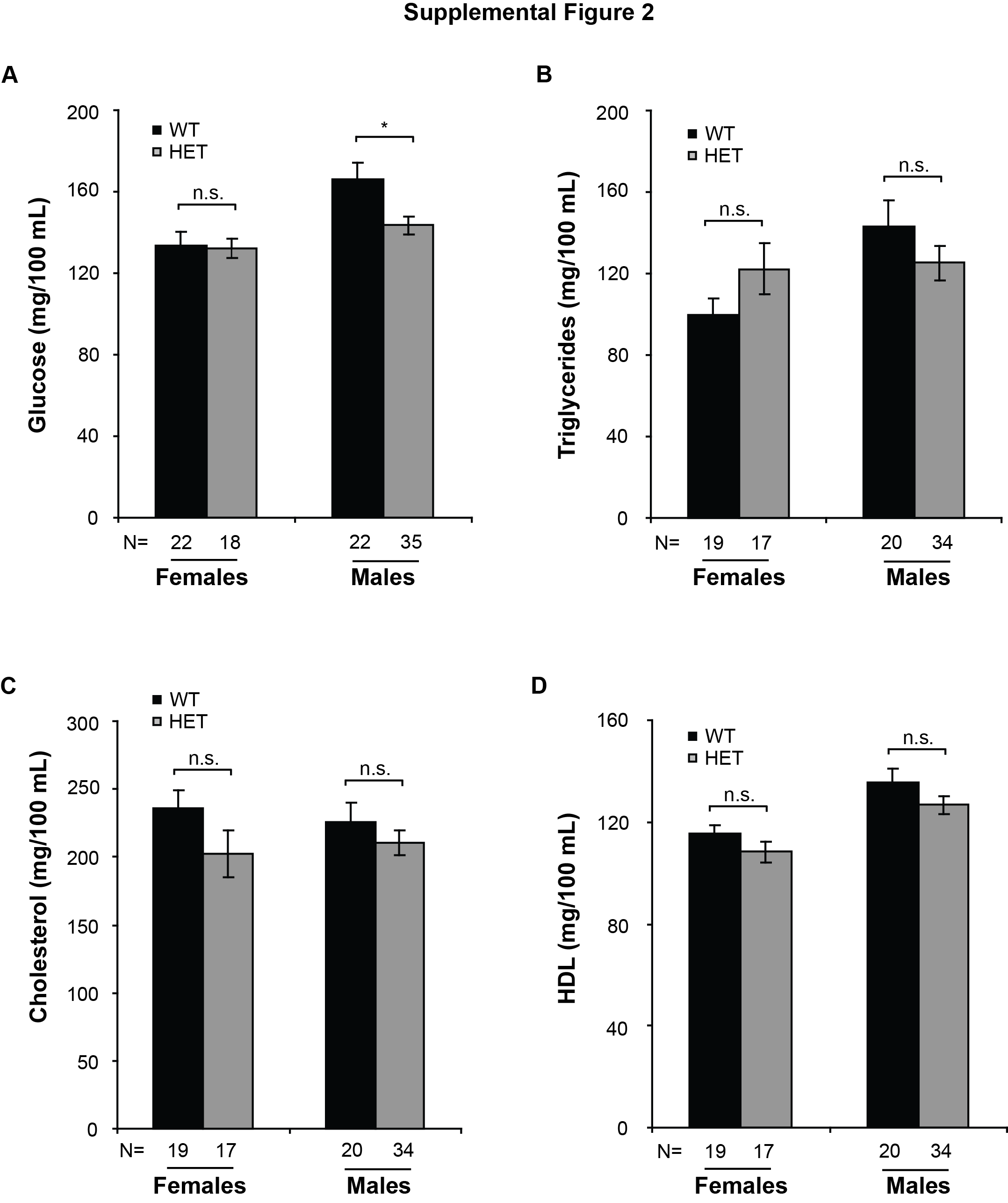

Supplement: Figure S2 — Assessment of additional metabolic parameters in Jll mice. Plasma was prepared from retro-orbital blood of wild-type (WT) and heterozygous Adcy3Jll/+ (HET) mice fed a HFD, and assessed for levels of other metabolically relevant markers. A modest decrease in fed glucose levels (A) was detectable in HET males, but not in females. No significant differences between WT and HET animals of either gender were observed for levels of triglycerides (B), total cholesterol (C) or HDL-cholesterol (D). All data are shown as mean +/− SEM, * p<0.05. (TIF) [file pone.0110226.s002.tif]

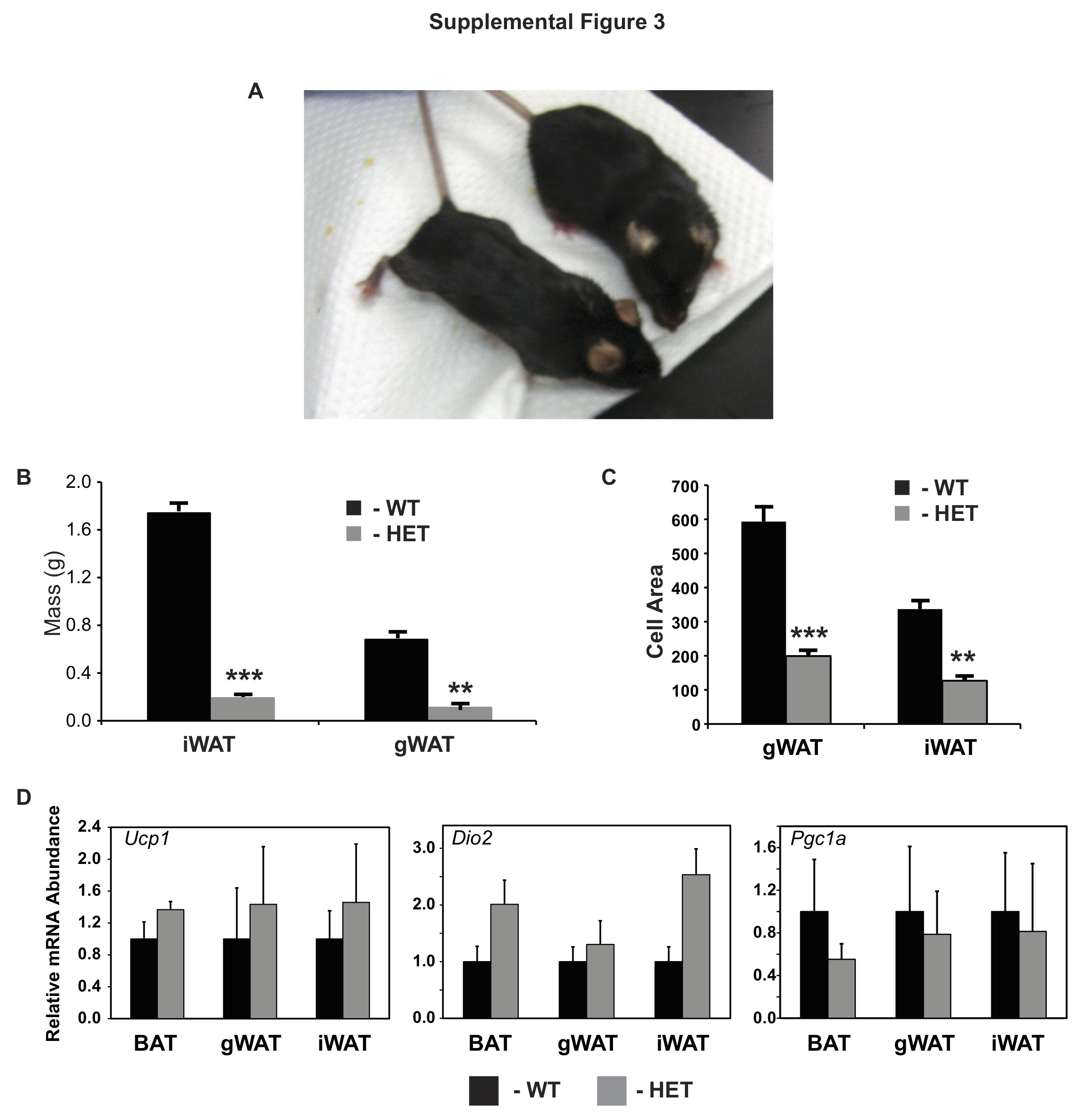

Supplement: Figure S3 — Jll protects mice from weight gain on HFD. A. Differences in size are visible between heterozygous Adcy3Jll/+ mice (left) and wild-type (right). B. Dissection and quantitation of white fat pad mass showed significantly less massive fat depots in heterozygous Adcy3Jll/+ (HET) mice relative to WT controls, both in gonadal (gWAT) and inguinal (iWAT) depots. C. Cross-sectional area of adipocytes from Adcy3Jll/+ (HET) and Adcy3+/+ (WT) mice. For each depot, area was measured with ImageJ software in four mice per genotype and two sections per mouse. Shown are the average ± S.E.M. for each genotype. D. Despite the reduction in adipocyte size in Jll mutant mice, no dramatic changes in the transcriptional levels of BAT-specific markers, such as Ucp1, Dio2, and Pgc1a, were detected in those adipose depots, N = 3 male mice per tissue per genotype. All data are shown as mean +/− SEM. For B and C, ** - p<0.01 and *** - p<0.001. (TIFF) [file pone.0110226.s003.tiff]
